# *In vivo* *(R)*-[^11^C]PK11195 PET imaging of 18kDa translocator protein in recent onset psychosis

**DOI:** 10.1038/npjschz.2016.31

**Published:** 2016-08-31

**Authors:** Thalia F van der Doef, Lot D de Witte, Arjen L Sutterland, Ellen Jobse, Maqsood Yaqub, Ronald Boellaard, Lieuwe de Haan, Jonas Eriksson, Adriaan A Lammertsma, René S Kahn, Bart N M van Berckel

**Affiliations:** 1 Department of Psychiatry, Rudolf Magnus Institute for Neurosciences, University Medical Center Utrecht, Utrecht, The Netherlands; 2 Department of Radiology & Nuclear Medicine, VU University Medical Center, Amsterdam, The Netherlands; 3 Department of Psychiatry, Academic Medical Center, Amsterdam, The Netherlands

## Abstract

Evidence is accumulating that immune dysfunction is involved in the pathophysiology of schizophrenia. It has been hypothesized that microglia activation is present in patients with schizophrenia. Various *in vivo* and post-mortem studies have investigated this hypothesis, but as yet with inconclusive results. Microglia activation is associated with elevations in 18 kDa translocator protein (TSPO) levels, which can be measured with the positron emission tomography (PET) tracer *(R)*-[^11^C]PK11195. The purpose of the present study was to investigate microglia activation in psychosis *in vivo* at an early stage of the disease. *(R)*-[^11^C]PK11195 binding potential (BP_ND_) was measured in 19 patients with recent onset psychosis and 17 age and gender-matched healthy controls. Total gray matter, as well as five gray matter regions of interest (frontal cortex, temporal cortex, parietal cortex, striatum, and thalamus) were defined *a priori*. PET data were analysed using a reference tissue approach and a supervised cluster analysis algorithm to identify the reference region. No significant difference in *(R)*-[^11^C]PK11195 BP_ND_ between patients and controls was found in total gray matter, nor one of the regions of interest. These findings suggest that microglia activation is not present in recent onset psychosis or that it is a subtle phenomenon that could not be detected using the design of the present study.

## Introduction

Schizophrenia is a complex and disabling disorder characterized by psychotic and cognitive symptoms, motivational impairment, and affective dysregulation. Its pathophysiology still remains to be elucidated. There is a growing body of evidence that immune dysfunction is involved in the pathophysiology of schizophrenia. Genetic data, derived from the latest and largest genome-wide association study, identified significant associations with immune pathways in schizophrenia.^1^ Studies using information from population registries have found increased prevalence of autoimmune disorders in patients with schizophrenia and their family members.^2^ Furthermore, decreased prevalence of schizophrenia has been reported in men who have used glucocorticoids for somatic disorders,^3^ and some studies suggest efficacy of immunomodulatory drugs in patients with schizophrenia with the largest effect found in first-episode patients.^4–6^ Serum and cerebrospinal fluid (CSF) inflammatory markers, such as cytokines produced by microglial cells, have also been linked with schizophrenia and are increased predominantly in first-episode psychosis.^7^

Although there is evidence for altered immune function in schizophrenia, it is still unclear which immune processes and immune cells are affected. Various studies using post-mortem tissue of schizophrenia patients and controls described increased density and alterations of microglia.^8,9^ These findings could not be replicated in other studies, which may be due to small sample sizes and post-mortem confounders.^8^ Moreover, post-mortem brain tissue of patients in the early phase of the disease is very scarce.

Altered microglia density and phenotype is associated with increased 18 kDa translocator protein (TSPO) expression. Until now, the only method to investigate microglia cells in the brain *in vivo* is positron emission tomography (PET) using TSPO ligands.^10^ In classic central nervous system (CNS) autoimmune disorders such as multiple sclerosis increased binding of TSPO ligands was demonstrated.^11^ However, full-blown neuroinflammation is less evident in schizophrenia.^12^ PET studies examining TSPO binding in patients with schizophrenia have provided inconclusive results. Using the TSPO ligand (*R*)-[^11^C]PK11195, studies have reported increased binding potential in schizophrenia patients in total gray matter^13^ and hippocampus.^14^ Recently, significantly increased distribution volume ratios were reported in total gray matter, and frontal and temporal cortex gray matter of patients with schizophrenia, and ultra-high-risk individuals when compared with controls using the tracer [^11^C]PBR28.^15^ Other studies using the second-generation TSPO PET ligands [^11^C]DPA-713, [^18^F]FEPPA, and [^11^C]DAA1106, however, did not find significant differences between patients and controls.^16–18^

Important reasons for these inconsistent findings using PET imaging could be differences in patients characteristics and PET methodology.^19^ For example, the disease duration of previous PET studies varied, as there was one ultra-high-risk assessment,^15^ two studies examined patients with schizophrenia within 5 years after disease onset,^13,16^ and other studies examined chronic patients.^14,15,17^ Clinical trials showed biggest effect of immunomodulatory drugs in the early stages of schizophrenia.^5,6^ The purpose of the present study was to examine *(R)*-[^11^C]PK11195 binding potential (BP_ND_) in total gray matter and several gray matter regions of interest in recent onset psychosis. By using patients with recent onset psychotic disorder and matched controls the role of confounders was minimized.

## Results

Demographics of patients with a recent onset psychotic disorder and healthy controls are shown in Table 1 and Supplementary Table 1. Patients displayed moderate symptoms at the time of PET scanning. The average disease duration as well as the average duration of treatment with antipsychotic medication was 1 year. Most patients were on antipsychotic medication at the time of the scanning, apart from 3 patients who were not taking medication and 1 patient who was antipsychotic naive. There were no significant differences in demographics between patients and controls. Although a significant difference in specific activity of *(R)*-[^11^C]PK11195 between groups was found, the injected mass of PK11195 did not differ between clinical groups, and no significant associations have been found between specific activity or injected mass and *(R)*-[^11^C]PK11195 BP_ND_ (data not shown). Moreover, all PET scans were performed at tracer level. Nicotine and cannabis use was not matched across groups, and was more prevalent in the patient group.

**Figure 1:**
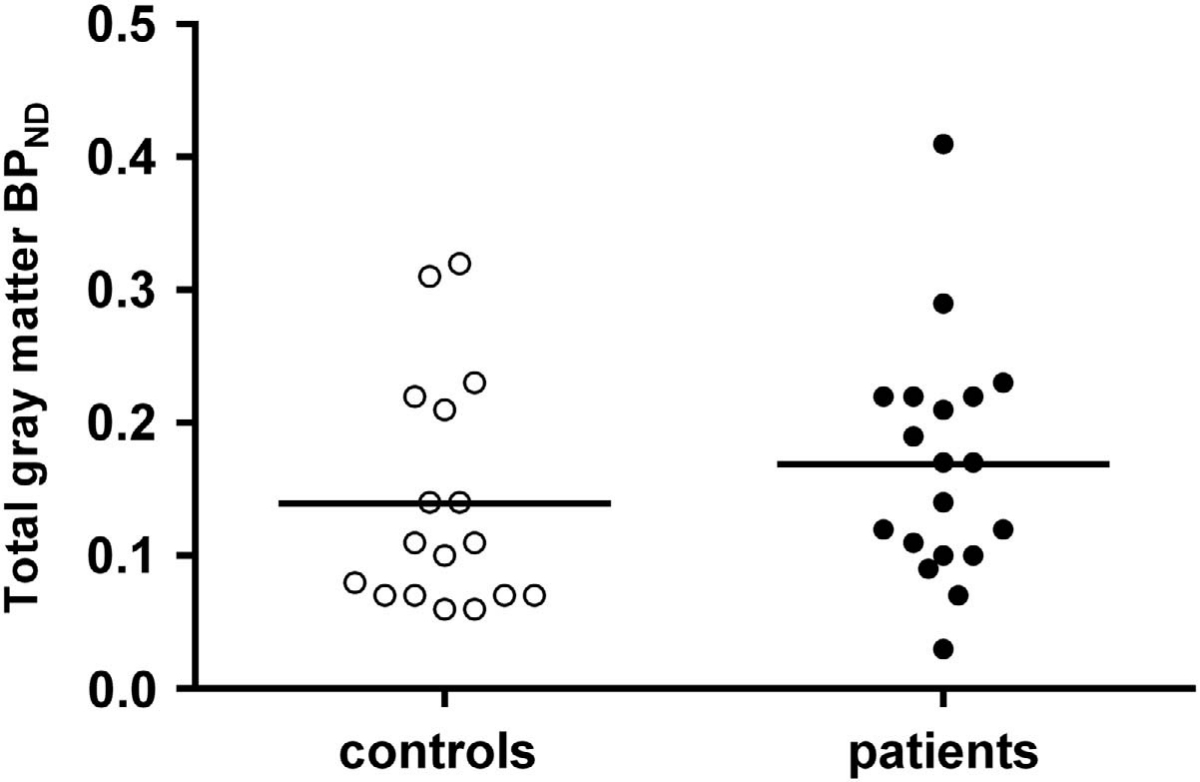
Scatterplot showing individual binding potential (BP_ND_) values of the total gray matter. No significant differences were found between patients with a recent onset psychotic disorder (black dots) compared with healthy controls (white dots). Bars indicate mean group BP_ND_ values.

**Figure 2:**
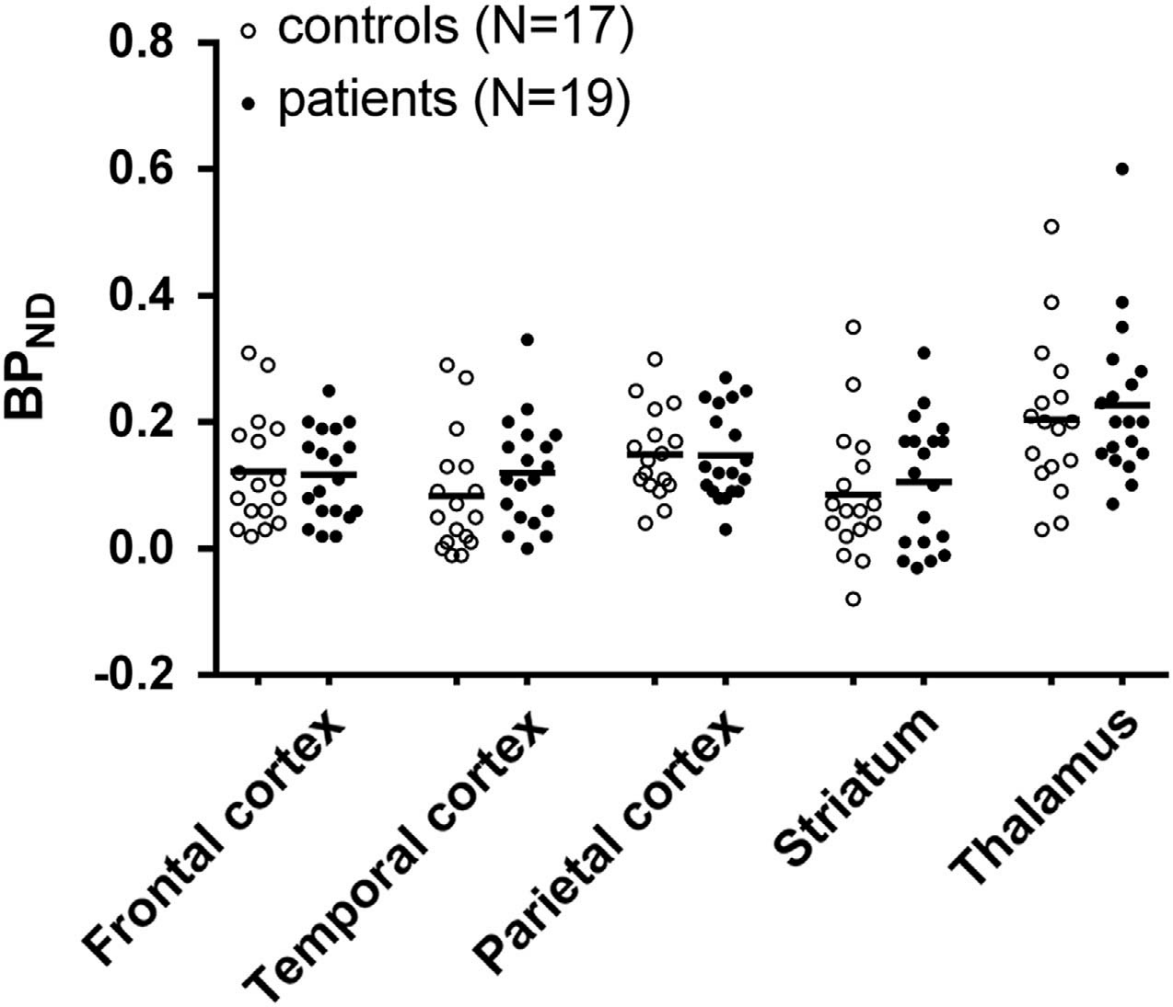
Scatterplot showing individual binding potential (BP_ND_) values in each of the studied regions of interest (frontal cortex, temporal cortex, parietal cortex, striatum, and thalamus). No significant differences were found between patients with a recent onset psychotic disorder (black dots) compared with healthy controls (white dots). Bars indicate mean group BP_ND_ values.

No significant difference in mean *(R)*-[^11^C]PK11195 BP_ND_ between groups was observed in total gray matter, either with or without age as covariate (Figure 1). Similarly, for regional *(R)*-[^11^C]PK11195 BP_ND_ no significant effect of clinical group was found (Figure 2, Table 2).

A significant correlation was found between age and total gray matter *(R)*-[^11^C]PK11195 BP_ND_ when all data were analysed (*N*=36, *r*=0.38, *P*=0.02; Supplementary Figure 2), as well as age and regional *(R)*-[^11^C]PK11195 BP_ND_ in all ROIs (*N*=36, *r*=0.72–0.95, *P*<0.01). No significant correlations were observed between total and regional gray matter *(R)*-[^11^C]PK11195 BP_ND_ and other variables, such as alcohol, nicotine or cannabis use, disease duration, and disease severity (Supplementary Table 2; correlations between *(R)*-[^11^C]PK11195 BP_ND_ and PANSS subscale scores are available upon request). *(R)*-[^11^C]PK11195 BP_ND_ did not significantly differ between patients on and off antipsychotics, nor was there a significant difference between antipsychotics (Supplementary Table 3).

## Discussion

In this study, microglia activation in gray matter ROIs of patients with a recent onset psychotic disorder were investigated *in vivo*. There was no significant difference in *(R)*-[^11^C]PK11195 BP_ND_ between patients and controls in total gray matter, nor in any of the ROIs investigated. In addition, no relationship was found between *(R)*-[^11^C]PK11195 BP_ND_ and clinical measurements, such as symptom severity.

This study is in line with previous negative findings of three PET studies examining patients with schizophrenia using the second-generation ligands [^11^C]DPA-713, [^18^F]FEPPA, and [^11^C]DAA1106, respectively,^16–18^ but is in contrast to two *(R)*-[^11^C]PK11195 studies that have reported significant increases in *(R)*-[^11^C]PK11195 binding in total gray matter^13^ and hippocampus^14^ in patients with schizophrenia, and to one [^11^C]PBR28 study that reported increased distribution volume ratios in total gray matter and frontal and temporal cortex gray matter of patients with schizophrenia and ultra-high-risk individuals.^15^

In this study, whole-brain ROIs were selected because of the widespread TSPO availability in the brain. Combined ROIs were selected to avoid type I errors, which resulted in the inclusion of the hippocampus in the temporal region. When examining the hippocampus alone, there was also no significant difference (data not shown). Previous PET studies that examined widespread brain ROIs in schizophrenia,^14^ found a localized effect in temporal and frontal ROIs only.^14,15^

There can be several explanations for the discrepancy across the TSPO PET studies. First of all, this discrepancy in findings may be due to the differences in disease duration. Microglia can shift in function and morphology during the course of the illness^20^ and may be involved in (later stages of) schizophrenia rather than being associated with recent onset psychosis.

The patients in the present study have a short disease duration, which resulted in the inclusion of patients with a psychotic disorder not (yet) fulfilling criteria for schizophrenia. Our findings are in line with a recent study that reported no differences distribution volume (*V*_T_) values in recent onset schizophrenia.^16^ In addition, no differences in *V*_T_ values were found in ultra-high-risk individuals using two-tissue compartmental model (2TCM).^15^ However, if analysed with a 2TCM accounting for endothelial vascular TSPO binding (2TCM-1K), this study did find an effect in ultra-high-risk individuals,^15^ and another *(R)*-[^11^C]PK11195 study found an effect in patients with an illness duration of less than first 5 years suggesting that microglia activation could be present in the early phase(s) of the disease.^13^

Second, a positive association between symptom severity and TSPO expression has been reported in some of the previous studies.^15,17^ This suggests that the level of symptomatology could be a reason for the discrepant results. However, as symptom severity in the present study was not different from that in previous PET studies reporting an effect, this seems unlikely.^13–17^

Third, general concerns with the inclusion of schizophrenia patients are antipsychotic treatment and substance use, such as nicotine and cannabis use. Indeed, it has been shown *in vitro* that TSPO expression is reduced in cell lines treated with most atypical antipsychotics, apart from clozapine, which tended to increase TSPO expression.^21^ In the present study, no significant differences between patients on or off antipsychotics were found, but subgroups were small. Future studies may be able to address this issue by examining antipsychotic naive patients, which so far has only been performed in ultra-high-risk individuals.^15^ In addition, nicotine and cannabis use might reduce TSPO expression, as they have a suppressant effect on the immune system.^22,23^ Nicotine and cannabis use was more prevalent in the patient group. However, no significant association between nicotine or cannabis use and *(R)*-[^11^C]PK11195 BP_ND_ was observed.

Furthermore, the differences might be explained by differences in methodology.^19^ *(R)*-[^11^C]PK11195 has the advantage that it has been extensively investigated due to more than 30 years of research.^24^ A limitation is its lower signal-to-noise ratio in comparison with second-generation tracers, which has been demonstrated by animal blocking studies showing up to 80-fold higher specific binding in second-generation ligands, such as [^11^C]PBR28 and [^18^F]FEPPA, compared with *(R)*-[^11^C]PK11195.^25–27^ In addition, biomathematical modeling of *in silico*/*in vitro* data indicated a higher predicted within-subject variability for *(R)*-[^11^C]PK11195 than second-generation ligands, such as [^11^C]PBR28 and [^11^C]DPA-713.^28^ Direct *in vivo* comparison between *(R)*-[^11^C]PK11195 and second-generation tracers in humans is limited, although the second-generation ligand [^11^C]DPA-713 showed higher *V*_T_ values in comparison to *(R)*-[^11^C]PK11195 in a small group (*N*=2) of healthy volunteers.^29^ The negative findings of the present study seemed not to be tracer specific as previous *(R)*-[^11^C]PK11195 have reported an effect,^13,14^ and second-generation tracers have reported both negative and positive findings.^15–18^ The two-tissue reversible plasma input model (2T4k) is the gold standard for analysis of dynamic *(R)*-[^11^C]PK11195 studies.^30^ However, plasma input models are more invasive than reference tissue methods. In general, there is no anatomical reference ROI available, as there is no brain region devoid of TSPO. If a reference region is not completely devoid of TSPO, this may result in underestimation of the signal. For instance, *(R)*-[^11^C]PK11195 may be affected by non-specific binding to endothelial tissue.^31^ However, no differences in time-activity curves of the reference region between patients and controls were found (data not shown). In addition, to avoid the effect of binding to venous sinuses, a vascular correction was applied in the present study.^31,32^

Lastly, a general problem is the large intersubject variability in TSPO expression, which is shown in this and other PET studies.^14–16^ To correct this variability, normalization to whole-brain outcome was used in two studies that did report an effect, either by the use of the whole-brain gray matter binding potential as a covariate to correct for global uptake^14^ or by calculating the distribution volume ratio, which is defined as the ratio of *V*_T_ in the ROI to *V*_T_ in the whole-brain.^15^ Interestingly, the present results are in line with the (non-normalized) *V*_T_ values of one study, as no difference in this outcome measure was found.^15^ It needs to be stressed that the intersubject variability in the present groups was probably too large to detect significant differences. Therefore, larger sample sizes may be needed to detect a possible effect between groups. The sample size was relatively large for a PET study and according to the sample size calculation of a previous data set,^13^ which was reanalysed using the same method as in the present study (TFvdD *et al.*, unpublished), it should have been large enough to detect a clinically significant difference using the present method. However, microglia activation might be a relatively subtle phenomenon in psychosis, which is not easily detected.

In the present study, no corrections for partial volume effects were applied, as no reduction of gray matter volume in patients was found, nor a correlation with BP_ND_ (data not shown). In addition, a previous TSPO study did not report differences between partial volume corrected and uncorrected data in psychosis, indicating that partial volume effects are minor.^17^

The inclusion of patients with a short disease duration is the main strength of the present study. A limitation is the fact that most patients were on (atypical) antipsychotics. A general limitation for *(R)*-[^11^C]PK11195 and other TSPO ligands is that the actual cellular process underlying the PET signal is unclear.^20^ TSPO ligands do not exclusively bind to microglia, but may also bind to other cells in the CNS, such as astrocytes.^33,34^ Furthermore, for the microglia population, TSPO ligands cannot distinguish between pro-inflammatory and anti-inflammatory subpopulations. Future studies may develop more specific microglia markers that are also sensitive to the functional phenotype.^19^

In conclusion, no evidence was found with increased microglial activation in gray matter in recent onset psychosis. These findings suggest that microglia activation is not present in recent onset psychosis or that it is a subtle phenomenon that could not be detected due to other factors, such as patient characteristics and antipsychotic treatment. Further studies, preferably using more sensitive TSPO tracers, are warranted to ascertain the role of microglia in the pathophysiology of schizophrenia.

## Materials and methods

### Subjects

Overall, 19 patients with a recent onset psychotic disorder and 17 age and sex-matched healthy controls were included. Patients were recruited from the Department of Psychiatry of the University Medical Center Utrecht (UMCU), Utrecht, The Netherlands, and the Department of Psychiatry of the Academic Medical Center (AMC), Amsterdam, The Netherlands. Controls were recruited via advertisements. Eligible patients were aged 18–40 years, met diagnostic criteria for a psychotic disorder, and were studied within the first 2 years of duration of treatment or were antipsychotic naive. Duration of treatment was defined as first intake of antipsychotic medication. DSM-IV diagnoses were confirmed by two investigators using the Comprehensive Assessment of Symptoms and History (CASH) interview.^35^ Exclusion criteria were any neurological disorder, pregnancy, abnormal score on routine laboratory tests (blood tests and urine analysis), intake of non-cannabis addictive drugs or use of benzodiazepines and non-steroidal anti-inflammatory drugs (NSAIDs) (⩽4 weeks before start of the study), and current and lifetime alcohol abuse.

Additional exclusion criteria for healthy controls were presence or history of psychiatric disorder confirmed by the CASH interview or a first-degree relative with a family history of schizophrenia spectrum disorder. The study complied with the Declaration of Helsinki and had been approved by the Medical Ethics Review Committee of UMCU. All subjects signed an informed consent form before inclusion.

Demographical data were collected within 1 week of performing the PET scan. Current and lifetime use of alcohol, nicotine, cannabis, and other illicit drugs was assessed using the Composite International Diagnostic Interview (CIDI)^36^ and disease severity with the positive and negative syndrome scale (PANSS).^37^ Complete blood count, serum chemistry, use of benzodiazepines and addictive drugs (3, 4-methylenedioxymethamphetamine, amphetamine, cannabis, cocaine, opiates), and pregnancy were tested with laboratory tests within 1 week of the PET scan.

### PET procedures

PET scans were performed on an Philips Gemini TF-64 positron emission tomography/computed tomography (PET/CT) scanner (Philips Medical Systems, Best, The Netherlands).^38^ Tracer administration was standardized. After administration of a bolus injection of 370 MBq *(R*)-[^11^C]PK11195 using an infusion pump (Med-Rad, Beek, The Netherlands), a dynamic three-dimensional (3D) emission scan of 60.5 min was acquired as described previously.^31^ Before the emission scan, a low-dose computed tomography (CT) scan was acquired on the PET/CT scanner, which was used to correct the subsequent PET scan for tissue attenuations. Using laser beams, subject motion during scanning was checked visually at regular intervals and corrected immediately, whenever necessary. Moreover, motion was minimized by using a head immobilization device (head holder). PET data were corrected for dead time, decay, tissue attenuation, scatter, and randoms. PET images were reconstructed using a dedicated brain mode iterative algorithm (LOR-RAMLA)^37^ and had a voxel size of 2×2×2 mm^3^.

### MRI scans

A structural T1-weighted magnetic resonance imaging (MRI) scan was acquired for each subject. Subjects were scanned on a Philips Achieva 3T scanner (Best, The Netherlands; voxel size 0.75×0.75×0.75 mm^3^). MRI scans were examined by a neuroradiologist to exclude major brain abnormalities in both patients and controls.

### Image analysis

PET data were analysed using a reference tissue approach as described previously.^31,32,39^ In short, this procedure uses supervised cluster analysis algorithm with four kinetic classes (SVCA4) to extract the reference tissue input function. SCVA4 first applies an anatomical mask to include brain tissue only and then the algorithm clusters voxels in the following predefined kinetic classes: gray matter with specific *(R*)-[^11^C]PK11195 binding, gray matter without specific binding, white matter, and blood. Gray matter voxels without specific binding were used as reference tissue input. Next, BP_ND_ parametric images of *(R*)-[^11^C]PK11195 scans were generated using reference parametric mapping with a vascular correction (RPM*V*_b_),^40^ which is a basis function implementation of the simplified reference tissue model.^41^

T1-weighted MRI scans were co-registered with PET scans and used for segmentation of gray and white matter. Then, the co-registered MRI scan was used to delineate total gray matter, and to define five gray matter ROIs including the frontal cortex (volume weighted average of anterior cingulate cortex, gyrus rectus, orbitofrontal cortex, precentral gyrus, superior frontal cortex, middle frontal cortex, interior frontal cortex), temporal cortex (volume weighted average of hippocampus, anterior and posterior temporal cortex, parahippocampal, and ambient cortex, superior, middle and inferior temporal cortex, fusiform cortex), parietal cortex (volume weighted average of posterior cingulate cortex, postcentral gyrus, superior parietal cortex, and inferolateral parietal cortex), thalamus and striatum (volume weighted average of caudate nucleus and putamen) using probability map-based automatic brain delineation.^42^ This selection of ROIs was based on the widespread TSPO availability in the brain. The outcome measure was *(R*)-[^11^C]PK11195 binding potential (BP_ND_; Supplementary Figure 1).^41,43^

### Statistics

Analysis of variance (ANOVA) and multivariate analysis of variance (MANOVA) with ROI as within-subject factor, group as between-subject factor and age as covariate were carried out to test for differences between groups (two-sided) in total gray matter and regional *(R*)-[^11^C]PK11195 BP_ND_, respectively. A subsequent MANOVA as a *post-hoc* analysis was implemented to identify regional group differences between patients and controls (two-sided). Homogeneity of variances was tested by the Levene's test for equality of variances and was not violated for *(R)*-[^11^C]PK11195 BP_ND_ in all regions tested.

Pearson’s correlation coefficient *(r)* was used to evaluate correlations between *(R*)-[^11^C]PK11195 BP_ND_ and other variables, such as demographics. The χ^2^ test was used to test between-group differences in gender. *P* values below 0.05 were considered to be significant and Bonferroni corrections were used for multiple comparisons. The power calculation was based on previous data,^13^ which were reanalysed using the same method as in the present study (using a reference tissue approach and a supervised cluster analysis algorithm to identify the reference region, TFvdD *et al*., unpublished). This resulted in the requirement of at least 16 subjects per group to detect a difference with 80% power to examine five ROIs (*μ*_1_=0.07, *μ*_2_=0.18, *σ*_1_=0.10, *σ*_2_=0.08, *α*=0.01).

## Figures and Tables

**Table 1:** Demographics

	*Controls *(N*=17*)	*Patients *(N*=19*)		
Age (years, (range))	26±4 (20–34)	26±4 (20–34)	*F=*0.01	*P=*0.95
Gender (male/female)	14/3	16/3	*χ*^2^*=*0.02	*P=*0.89
Injected dose (MBq)	433±23	407±73	*F=*1.71	*P=*0.20
Specific activity (GBq/μmol)	96±28	74±19	*F=*6.64	*P=*0.02
Injected mass (μg)	1.7±0.6	2.1±0.6	*F=*2.44	*P=*0.13
Nicotine (user/non-user)	5/12	13/6		
Cannabis (positive/negative)	0/17	4/15		
Duration of illness (years)		1.3±1.1		
				
*PANSS*				
Total		53±10		
Positive		12±4		
Negative		14±3		
General		27±6		
				
*Antipsychotics*				
None		4		
Clozapine		3		
Risperidone		2		
Olanzapine		5		
Other		5		

Abbreviation: PANSS, positive and negative syndrome scale.

Values are presented as mean±s.d.

**Table 2:** *(R)*-[^11^C]PK11195 binding potential (BP_ND_)

*ROI*	*Controls *(N*=17*)	*Patients *(N*=19*)	*F*	P
Total gray matter	0.14±0.09	0.17±0.09	1.11	0.30
Frontal cortex	0.12±0.09	0.12±0.07	0.03	0.85
Temporal cortex	0.08±0.09	0.12±0.08	1.88	0.18
Parietal cortex	0.15±0.07	0.15±0.07	0.00	0.97
Striatum	0.09±0.10	0.10±0.10	0.41	0.53
Thalamus	0.20±0.12	0.23±0.12	0.40	0.53

Abbreviation: ROI, region of interest.

Values are presented as mean±s.d.

## References

[bib1] Network and Pathway Analysis Subgroup of Psychiatric Genomics Consortium. Psychiatric genome-wide association study analyses implicate neuronal, immune and histone pathways. Nat. Neurosci. 18, 199–209 (2015). 25599223 10.1038/nn.3922 PMC4378867

[bib2] Benros, M. E. et al. Autoimmune diseases and severe infections as risk factors for schizophrenia: a 30-year population-based register study. Am. J. Psychiatry 168, 1303–1310 (2011). 22193673 10.1176/appi.ajp.2011.11030516

[bib3] Laan, W. et al. Glucocorticosteroids associated with a decreased risk of psychosis. J. Clin. Psychopharmacol. 29, 288–290 (2009). 19440085 10.1097/JCP.0b013e3181a44575

[bib4] Laan, W. et al. Adjuvant aspirin therapy reduces symptoms of schizophrenia spectrum disorders: results from a randomized, double-blind, placebo-controlled trial. J. Clin. Psychiatry 71, 520–527 (2010). 20492850 10.4088/JCP.09m05117yel

[bib5] Sommer, I. E. et al. Efficacy of anti-inflammatory agents to improve symptoms in patients with schizophrenia: an update. Schizophr. Bull. 40, 181–191 (2014). 10.1093/schbul/sbt139 PMC3885306 24106335

[bib6] Nitta, M. et al. Adjunctive use of nonsteroidal anti-inflammatory drugs for schizophrenia: a meta-analytic investigation of randomized controlled trials. Schizophr. Bull. 39, 1230–1241 (2013). 23720576 10.1093/schbul/sbt070 PMC3796088

[bib7] Miller, B. J., Buckley, P., Seabolt, W., Mellor, A. & Kirkpatrick, B. Meta-analysis of cytokine alterations in schizophrenia: clinical status and antipsychotic effects. Biol. Psychiatry 70, 663–671 (2011). 21641581 10.1016/j.biopsych.2011.04.013 PMC4071300

[bib8] Bernstein, H. G., Steiner, J., Guest, P. C., Dobrowolny, H. & Bogerts, B. Glial cells as key players in schizophrenia pathology: recent insights and concepts of therapy. Schizophr. Res. 161, 4–18 (2014). 24948484 10.1016/j.schres.2014.03.035

[bib9] Fillman, S. G. et al. Increased inflammatory markers identified in the dorsolateral prefrontal cortex of individuals with schizophrenia. Mol. Psychiatry 18, 206–214 (2013). 22869038 10.1038/mp.2012.110

[bib10] Venneti, S., Lopresti, B. J. & Wiley, C. A. Molecular imaging of microglia/macrophages in the brain. Glia 61, 10–23 (2013). 22615180 10.1002/glia.22357 PMC3580157

[bib11] Doorduin, J., de Vries, E. F., Dierckx, R. A. & Klein, H. C. PET imaging of the peripheral benzodiazepine receptor: monitoring disease progression and therapy response in neurodegenerative disorders. Curr. Pharm. Des. 14, 3297–3315 (2008). 19075709 10.2174/138161208786549443

[bib12] Harrison, P. J. The neuropathology of schizophrenia. A critical review of the data and their interpretation. Brain 122, 593–624 (1999). 10219775 10.1093/brain/122.4.593

[bib13] van Berckel, B. N. et al. Microglia activation in recent-onset schizophrenia: a quantitative (R)-[11C]PK11195 positron emission tomography study. Biol. Psychiatry 64, 820–822 (2008). 18534557 10.1016/j.biopsych.2008.04.025

[bib14] Doorduin, J. et al. Neuroinflammation in schizophrenia-related psychosis: a PET study. J. Nucl. Med. 50, 1801–1807 (2009). 19837763 10.2967/jnumed.109.066647

[bib15] Bloomfield, P. S. et al. Microglial activity in people at ultra high risk of psychosis and in schizophrenia: an [^11^C]PBR28 PET brain imaging study. Am. J. Psychiatry 173, 44–52 (2015). 26472628 10.1176/appi.ajp.2015.14101358 PMC4821370

[bib16] Coughlin, J. M. et al. *In vivo* markers of inflammatory response in recent-onset schizophrenia: a combined study using [^11^C]DPA-713 PET and analysis of CSF and plasma. Transl. Psychiatry 12, e777 (2016). 10.1038/tp.2016.40 PMC4872398 27070405

[bib17] Kenk, M. et al. Imaging neuroinflammation in gray and white matter in schizophrenia: an in-vivo PET study with [18F]-FEPPA. Schizophr. Bull. 41, 85–93 (2015). 25385788 10.1093/schbul/sbu157 PMC4266311

[bib18] Takano, A. et al. Peripheral benzodiazepine receptors in patients with chronic schizophrenia: a PET study with [11C]DAA1106. Int. J. Neuropsychopharmacol. 13, 943–950 (2010). 20350336 10.1017/S1461145710000313

[bib19] van der Doef T. F., Doorduin J., van Berckel B. N., Cervenka S.. Assessing brain immune activation in psychiatric disorders: clinical and preclinical PET imaging studies of the 18-kDa translocator protein. Clin. Transl. Imaging 3, 449–460 (2015). 10.1007/s40336-015-0140-0 PMC5496979 28781965

[bib20] Harry, G. J. Microglia during development and aging. Pharmacol. Ther. 139, 313–326 (2013). 23644076 10.1016/j.pharmthera.2013.04.013 PMC3737416

[bib21] Danovich, L. et al. The influence of clozapine treatment and other antipsychotics on the 18 kDa translocator protein, formerly named the peripheral-type benzodiazepine receptor, and steroid production. Eur. Neuropsychopharmacol. 18, 24–33 (2008). 17561380 10.1016/j.euroneuro.2007.04.005

[bib22] Guan, Y.-Z. et al. Nicotine inhibits microglial proliferation and is neuroprotective in global ischemia rats. Mol. Neurobiol. 51, 1480–1488 (2015). 25095782 10.1007/s12035-014-8825-3

[bib23] Suárez-Pinilla, P., López-Gil, J. & Crespo-Facorro, B. Immune system: a possible nexus between cannabinoids and psychosis. Brain Behav. Immun. 40, 269–282 (2014). 24509089 10.1016/j.bbi.2014.01.018

[bib24] Liu, G. J. et al. The 18 kDa translocator protein, microglia and neuroinflammation. Brain Pathol. 24, 631–653 (2014). 25345894 10.1111/bpa.12196 PMC8029074

[bib25] Kreisl, W. C. et al. Comparison of [(11)C]-(R)-PK 11195 and [(11)C]PBR28, two radioligands for translocator protein (18 kDa) in human and monkey: implications for positron emission tomographic imaging of this inflammation biomarker. Neuroimage 49, 2924–2932 (2010). 19948230 10.1016/j.neuroimage.2009.11.056 PMC2832854

[bib26] Imaizumi, M. et al. Brain and whole-body imaging in nonhuman primates of [11C]PBR28, a promising PET radioligand for peripheral benzodiazepine receptors. Neuroimage 39, 1289–1298 (2008). 18024084 10.1016/j.neuroimage.2007.09.063 PMC2275117

[bib27] Rusjan, P. M. et al. Quantitation of translocator protein binding in human brain with the novel radioligand [18F]-FEPPA and positron emission tomography. J. Cereb. Blood Flow Metab. 31, 1807–1816 (2011). 10.1038/jcbfm.2011.55 PMC3170950 21522163

[bib28] Guo, Q., Owen, D. R., Rabiner, E. A., Turkheimer, F. E. & Gunn, R. N. Identifying improved TSPO PET imaging probes through biomathematics: the impact of multiple TSPO binding sites in vivo. Neuroimage 60, 902–910 (2012). 22251896 10.1016/j.neuroimage.2011.12.078 PMC3314937

[bib29] Endres, C. J. et al. Initial evaluation of 11C-DPA-713, a novel TSPO PET ligand, in humans. J. Nucl. Med. 50, 1276–1282 (2009). 19617321 10.2967/jnumed.109.062265 PMC2883612

[bib30] Gunn, R. N., Gunn, S. R. & Cunningham, V. J. Positron emission tomography compartmental models. J. Cereb. Blood Flow Metab. 21, 635–652 (2001). 11488533 10.1097/00004647-200106000-00002

[bib31] Schuitemaker, A. et al. Microglial activation in healthy aging. Neurobiol. Aging 33, 1067–1072 (2012). 21051106 10.1016/j.neurobiolaging.2010.09.016

[bib32] Yaqub, M. et al. Optimization of supervised cluster analysis for extracting reference tissue input curves in (R)-[11C]PK11195 brain PET studies. J. Cereb. Blood Flow Metab. 32, 1600–1608 (2012). 22588187 10.1038/jcbfm.2012.59 PMC3421099

[bib33] Tóth, M. et al. Acute neuroinflammation in a clinically relevant focal cortical ischemic stroke model in rat: longitudinal positron emission tomography and immunofluorescent tracking. Brain Struct. Funct. 221, 1279–1290 (2015). 25601153 10.1007/s00429-014-0970-y

[bib34] Lavisse, S. et al. Reactive astrocytes overexpress TSPO and are detected by TSPO positron emission tomography imaging. J. Neurosci. 32, 10809–10818 (2012). 22875916 10.1523/JNEUROSCI.1487-12.2012 PMC6621018

[bib35] Andreasen, N. C., Flaum, M. & Arndt, S. The comprehensive assessment of symptoms and history (CASH). An instrument for assessing diagnosis and psychopathology. Arch. Gen. Psychiatry 49, 615–623 (1992). 1637251 10.1001/archpsyc.1992.01820080023004

[bib36] Kessler, R. C. et al. Clinical calibration of DSM-IV diagnoses in the World Mental Health (WMH) version of the World Health Organization (WHO) composite international diagnostic interview (WMH-CIDI). Int. J. Methods Psychiatr. Res. 13, 122–139 (2004). 15297907 10.1002/mpr.169 PMC6878301

[bib37] Kay, S. R., Fiszbein, A. & Opler, L. A. The positive and negative syndrome scale for schizophrenia. Schizophr. Bull. 13, 261–276 (1987). 3616518 10.1093/schbul/13.2.261

[bib38] Surti, S. et al. Performance of Philips Gemini TF PET/CT scanner with special consideration for its time-of-flight imaging capabilities. J. Nucl. Med. 48, 471–480 (2007). 17332626

[bib39] Tomasi, G. et al. Novel reference region model reveals increased microglial and reduced vascular binding of 11C-(R)-PK11195 in patients with Alzheimer’s disease. J. Nucl. Med. 49, 1249–1256 (2008). 18632810 10.2967/jnumed.108.050583

[bib40] Gunn, R. N., Lammertsma, A. A., Hume, S. P. & Cunningham, V. J. Parametric imaging of ligand-receptor binding in PET using a simplified reference region model. Neuroimage 6, 279–287 (1997). 9417971 10.1006/nimg.1997.0303

[bib41] Lammertsma, A. A. & Hume, S. P. Simplified reference tissue model for PET receptor studies. Neuroimage 4, 153–158 (1996). 9345505 10.1006/nimg.1996.0066

[bib42] Hammers, A. et al. Implementation and application of a brain template for multiple volumes of interest. Hum. Brain Mapp. 15, 165–174 (2002). 11835607 10.1002/hbm.10016 PMC6871918

[bib43] Innis, R. B. et al. Consensus nomenclature for in vivo imaging of reversibly binding radioligands. J. Cereb. Blood Flow Metab. 27, 1533–1539 (2007). 17519979 10.1038/sj.jcbfm.9600493

