# Supplementary material for: In vivo (R)-[11C]PK11195 PET imaging of 18kDa translocator protein in recent onset psychosis
Source: NPJ Schizophr. 2016 Aug 31;2:16031–. doi: 10.1038/npjschz.2016.31 (PMC5007116; doi:10.1038/npjschz.2016.31)
Supplement: Supplementary Information [file npjschz201631-s1.doc]

**SUPPLEMENTAL DATA**

**Supplemental Table 1** Detailed demographics of patients
**Supplemental Table 2** Pearson’s correlation analyses between *(R)*-[^11^C]PK11195 BP_ND_ and PANSS score, disease duration, and substance use in patients (N=19)
**Supplemental Table 3** Antipsychotic use and *(R)*-[^11^C]PK11195 BP_ND_ in patients (N=19)
**Supplemental Figure 1.** A typical example is shown of a RPM*V_b_* BP_ND_ image (A), corresponding T1-weighted MRI image (B) and probability map of a supervised cluster reference region (C) of a patient.
**Supplemental Figure 2.** Pearson’s correlation coefficient (r) is shown between total grey matter BP_ND_ and age for all data (N=36;A), healthy controls (N=17;B), and patients (N=19;C).

| **Supplemental Table 1** Detailed demographics of patients | | | | |  |  |  |
| --- | --- | --- | --- | --- | --- | --- | --- |
| Number | Age | Gender | Antipsychotics | DSM-IV | Episodes | Disease duration | Duration of treatment |
|  | *(years)* | *(male/female)* |  |  |  | *(years)* | *(years)* |
| 1 | 25 | M | zuclopentixole | 298.9 | 1 | 1.4 | 1.4 |
| 2 | 20 | M | olanzapine | 295.1 | 2 | 1.9 | 1.9 |
| 3 | 25 | M | aripiprazole | 295.3 | 1 | 0.4 | 0.4 |
| 4 | 31 | F | flupentixol, paliperidone | 295.3 | 2 | 2.2 | 2.2 |
| 5 | 23 | M | clozapine | 295.7 | 1 | 0.9 | 0.8 |
| 6 | 27 | M | olanzapine | 295.9 | 1 | 0.8 | 0.6 |
| 7 | 24 | M | olanzapine | 298.9 | 1 | 0.5 | 0.5 |
| 8 | 28 | M | medication free | 298.9 | 1 | 1.2 | 1.2 |
| 9 | 34 | F | quetiapine, penfluridon | 295.1 | 1 | 1.2 | 1.2 |
| 10 | 21 | F | quetiapine | 295.9 | 1 | 1.5 | 0.8 |
| 11 | 26 | M | olanzapine | 298.9 | 1 | 0.3 | 0.3 |
| 12 | 21 | M | clozapine | 295.9 | 1 | 2.3 | 1.8 |
| 13 | 23 | M | risperidone | 295.1 | 1 | 1.2 | 1.2 |
| 14 | 30 | M | medication free | 295.4 | 1 | 1.8 | 1.4 |
| 15 | 21 | M | clozapine | 295.3 | 2 | 0.8 | 0.8 |
| 16 | 29 | M | medication free | 295.3 | 1 | 0.5 | 0.5 |
| 17 | 25 | M | medication naive | 295.3 | 1 | 0.1 | 0.0 |
| 18 | 30 | M | risperidone | 295.9 | 2 | 4.8 | 1.7 |
| 19 | 26 | M | olanzapine | 298.8 | 1 | 0.3 | 0.3 |

**Supplemental Table 2** Pearson’s correlation analyses between *(R)*-[^11^C]PK11195 BP_ND_ and PANSS score, duration of treatment, and substance use in patients (N=19)

| ROI | PANSS | | Duration of treatment | | Substance use | | | | | |
| --- | --- | --- | --- | --- | --- | --- | --- | --- | --- | --- |
|  | *Total score* | |  | | *Alcohol* | | *Nicotine* | | *Cannabis* | |
|  | *r* | *p* | *r* | *p* | *r* | *p* | *r* | *p* | *r* | *p* |
| Total grey matter | -0.05 | 0.84 | 0.07 | 0.77 | -0,24 | 0,32 | -0,11 | 0,65 | 0,34 | 0,15 |
| Frontal cortex | 0.19 | 0.44 | 0.07 | 0.77 | -0,45 | 0,05 | -0,35 | 0,15 | 0,10 | 0,69 |
| Temporal cortex | 0.00 | 1.00 | 0.11 | 0.64 | -0,25 | 0,30 | -0,08 | 0,76 | 0,23 | 0,35 |
| Parietal cortex | 0.06 | 0.82 | 0.10 | 0.68 | -0,40 | 0,09 | -0,21 | 0,38 | 0,18 | 0,47 |
| Striatum | -0.15 | 0.53 | 0.09 | 0.71 | -0,01 | 0,96 | -0,21 | 0,38 | 0,37 | 0,11 |
| Thalamus | 0.06 | 0.82 | 0.14 | 0.58 | -0,19 | 0,44 | -0,02 | 0,95 | 0,45 | 0,05 |

PANSS, Positive and Negative Syndrome Scale; ROI, region of interest

**Supplemental Table 3** Antipsychotic use and *(R)*-[^11^C]PK11195 BP_ND_ in patients (N=19)

| Antipsychotics | | Total grey matter | Frontal cortex | Temporal cortex | Parietal cortex | Striatum | Thalamus |
| --- | --- | --- | --- | --- | --- | --- | --- |
| None | (N=4) | 0.20 ± 0.15 | 0.11 ± 0.07 | 0.12 ± 0.15 | 0.13 ± 0.07 | 0.14 ± 0.11 | 0.23 ± 0.11 |
| Clozapine | (N=3) | 0.14 ± 0.08 | 0.09 ± 0.06 | 0.10 ± 0.05 | 0.14 ± 0.10 | 0.13 ± 0.07 | 0.20 ± 0.07 |
| Risperidone | (N=2) | 0.16 ± 0.18 | 0.14 ± 0.17 | 0.12 ± 0.14 | 0.15 ± 0.17 | 0.15 ± 0.22 | 0.35 ± 0.36 |
| Olanzapine | (N=5) | 0.17 ± 0.06 | 0.11 ± 0.07 | 0.12 ± 0.08 | 0.15 ± 0.04 | 0.08 ± 0.07 | 0.22 ± 0.06 |
| Other | (N=5) | 0.16 ± 0.05 | 0.14 ± 0.06 | 0.13 ± 0.04 | 0.16 ± 0.08 | 0.07 ± 0.11 | 0.20 ± 0.11 |
|  |  | *F=0.14, p=0.97* | *F=0.21, p=0.93* | *F=0.06, p=0.99* | *F=0.10, p=0.98* | *F=0.49, p=0.74* | *F=0.52, p=0.72* |
| Values are presented as mean ± SD | | |  |  |  |  |  |


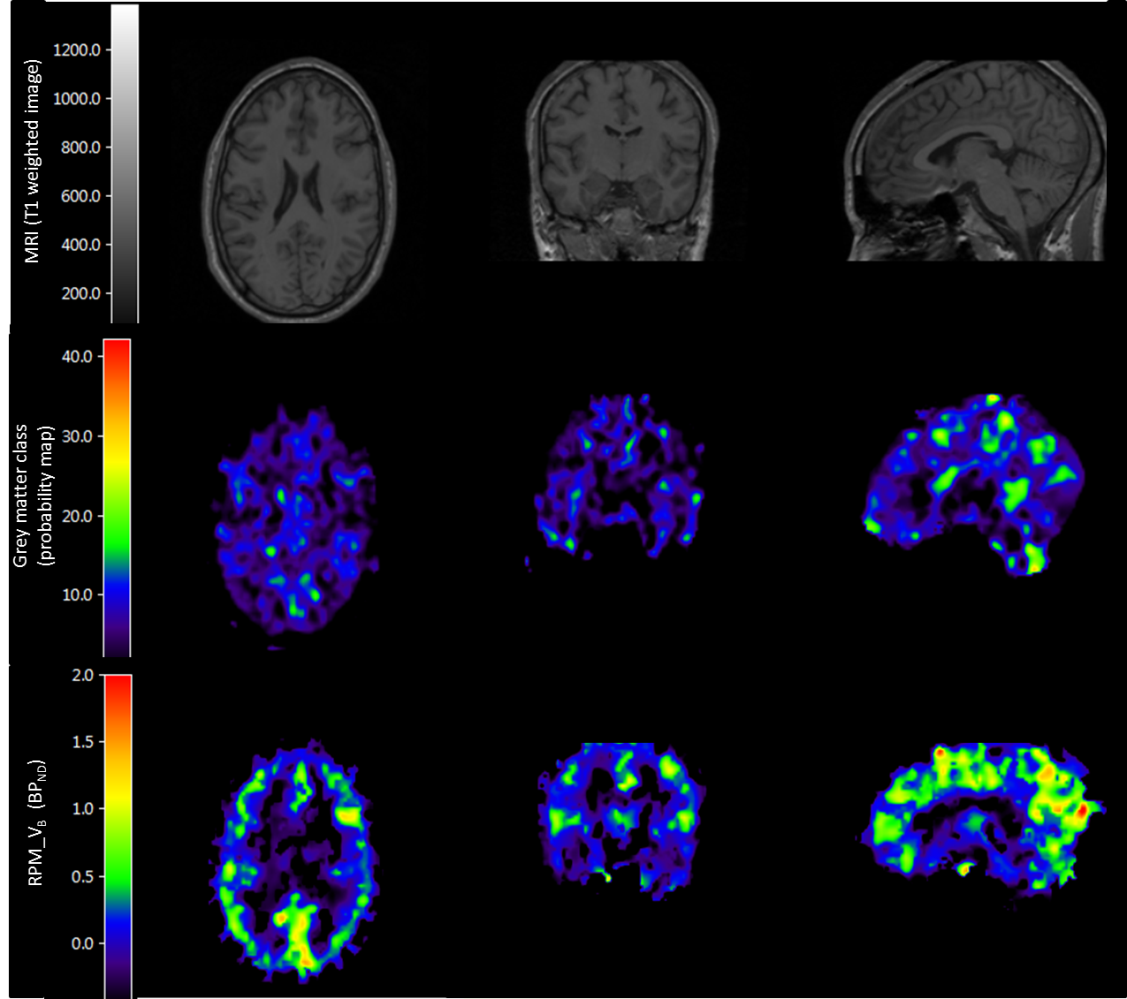


**Supplemental Figure 1.** A typical example is shown of a RPM*V_b_* BP_ND_ image (A), corresponding T1-weighted MRI image (B) and probability map of a supervised cluster reference region (C) of a patient. Representative axial, coronal, and sagittal slices.


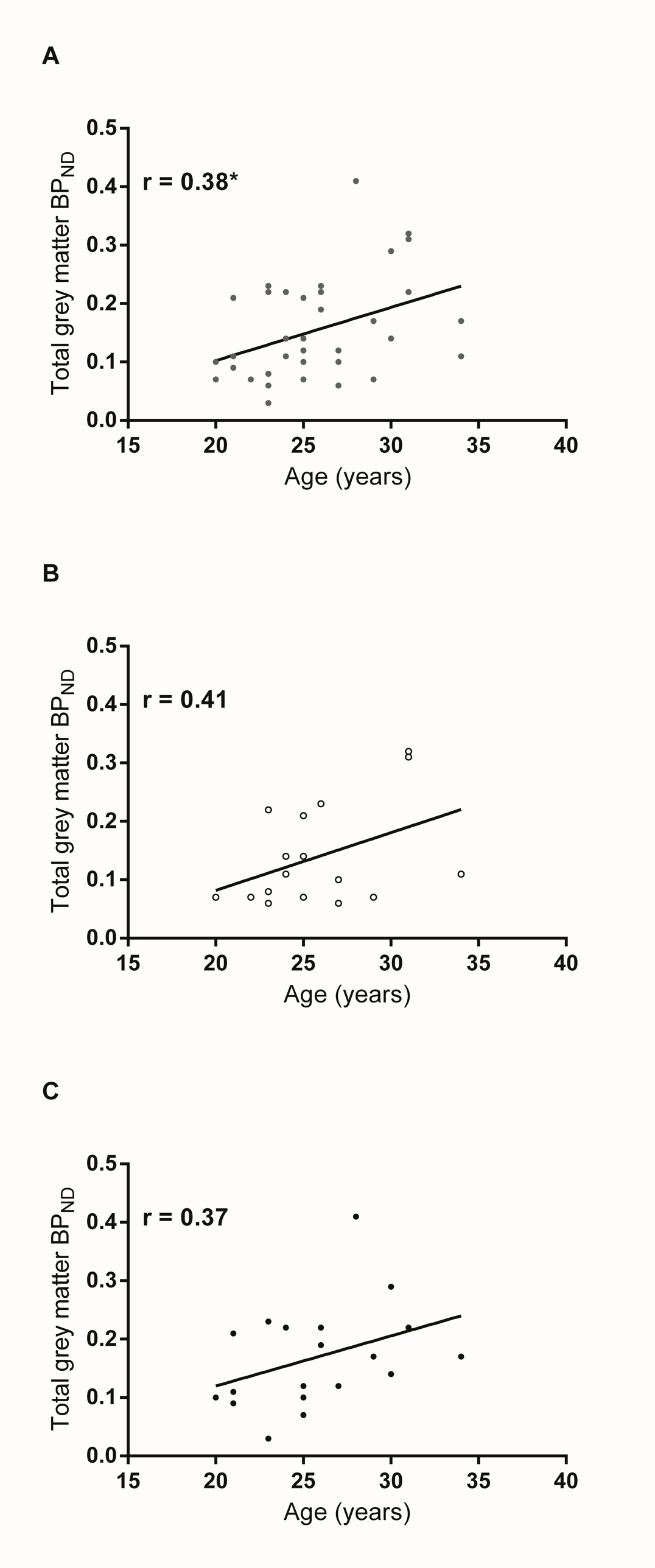


**Supplemental Figure 2.** Pearson’s correlation coefficient (r) is shown between total grey matter BP_ND_ and age for all data (N=36;A), healthy controls (N=17;B), and patients (N=19;C). A significant association between total grey matter BP_ND_ and age was found when all data were analysed (A; N=36, *r*=0.38, *p*=0.02), but no significant associations were observed for both groups separately (B, C).
