# Supplementary figures and images for: In vivo (R)-[11C]PK11195 PET imaging of 18kDa translocator protein in recent onset psychosis
Source: NPJ Schizophr. 2016 Aug 31;2:16031–. doi: 10.1038/npjschz.2016.31 (PMC5007116; doi:10.1038/npjschz.2016.31)

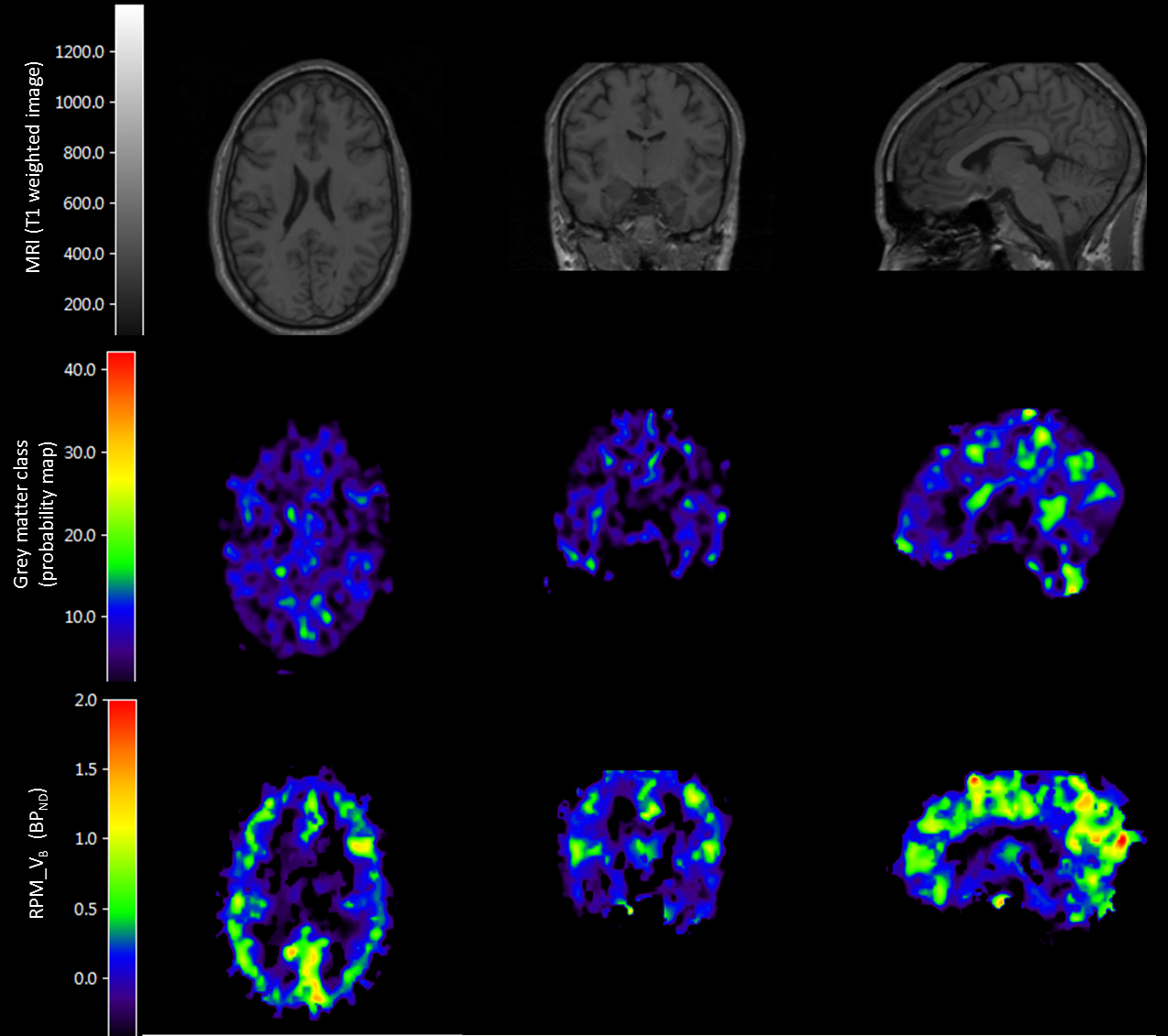

Supplement: Supplementary Figure 1 [file npjschz201631-s2.jpg]

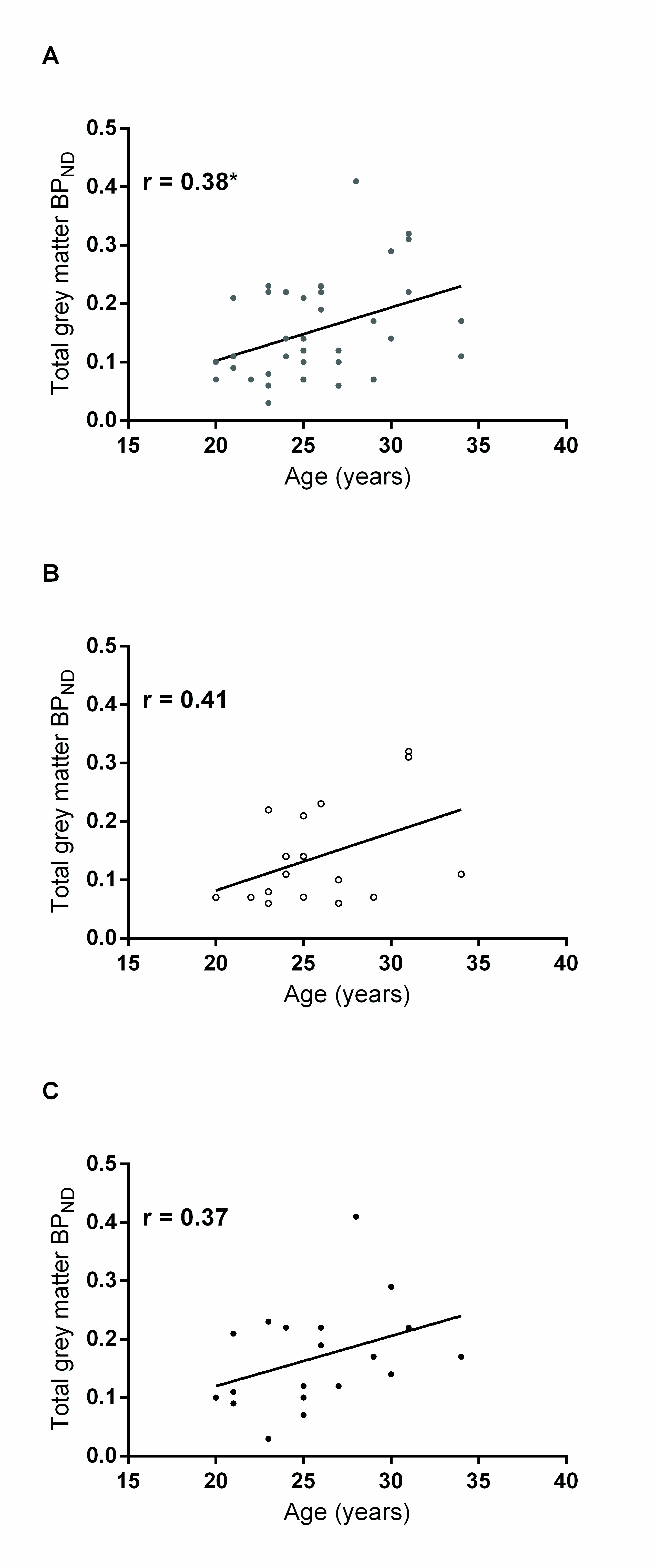

Supplement: Supplementary Figure 2 [file npjschz201631-s3.jpg]
